# Assessment of diagnostic reasoning in acute vertigo using vignette-based tools: A cross-sectional comparison between general practitioners and final-year medical students

**DOI:** 10.1371/journal.pone.0347129

**Published:** 2026-07-15

**Authors:** Charles Maquet, Maxime Wojtecki, Alexandre Tendron, Sophie Deneuve, Honorine Claudot, Frederic Crampon, Julien Burel

**Affiliations:** 1 Univ Rouen Normandie, Normandie Univ, GRHVN UR3830, CHU Rouen, Department of Otorhinolaryngology–Head and Neck Surgery, Rouen, France; 2 CHU Rouen, Department of General Practice and Primary Care, Rouen, France; 3 CHU Rouen, Department of Otorhinolaryngology–Head and Neck Surgery, Rouen, France; 4 Univ Rouen Normandie, Normandie Univ, Inserm U1245 and CHU Rouen, Department of Radiology, Rouen, France; UFPE: Universidade Federal de Pernambuco, BRAZIL

## Abstract

**Background:**

Acute vertigo is a frequent reason for consultation in primary care and poses diagnostic challenges for general practitioners. Although vestibular disorders are addressed during undergraduate medical training, knowledge retention over time is uncertain, while clinical experience accumulates with practice and may contribute to refining diagnostic reasoning. However, how diagnostic reasoning in acute vertigo evolves between the end of initial medical training and routine general practice remains poorly understood. Validated vignette-based tools, such as Script Concordance Tests and Key Feature Problems, enable standardized assessment of diagnostic reasoning. This study compares diagnostic reasoning in acute vertigo between final-year medical students and practicing general practitioners.

**Methods and findings:**

This cross-sectional observational study was conducted between January and March 2024 in a French region. Participants included general practitioners (GPs) in active practice and final-year medical students. All participants completed a self-administered questionnaire based on standardized clinical vignettes representing common presentations of acute vertigo. Diagnostic reasoning was assessed using two validated educational tools: Script Concordance Tests and Key Feature Problems. Scoring keys were established through consensus among 20 expert otolaryngologists. Diagnostic reasoning was expressed as the percentage of correct responses. Associations between diagnostic reasoning and participant characteristics, including clinical experience, consultation volume, vertigo-specific continuing medical education, and self-reported confidence in vertigo management, were analyzed using univariate and multivariate models. A total of 265 participants were included, comprising 173 general practitioners and 92 final-year medical students. Medical students achieved significantly higher diagnostic reasoning scores than GPs (61.0% vs. 54.0%, p < 0.001). Among GPs, higher scores were associated with lower daily consultation volumes (<30 patients/day), prior vertigo-specific continuing medical education, and greater self-reported confidence in the management of acute vertigo. In multivariate analysis, vertigo-specific continuing medical education (β = 0.77; 95% CI, 0.03–1.51; p = 0.041) and self-reported confidence (β = 1.45; 95% CI, 0.54–2.36; p = 0.002) remained independently associated with better diagnostic reasoning.

**Conclusion:**

In this study, final-year medical students outperformed practicing general practitioners on vignette-based assessments of diagnostic reasoning in acute vertigo. The observed difference may be related to differences in recent theoretical exposure and training background. Targeted continuing medical education could be further evaluated as a strategy to support diagnostic reasoning and confidence among general practitioners in primary care.

## Introduction

Vertigo is a frequent complaint in primary care, affecting 15% to 35% of individuals over their lifetime [1]. It encompasses a broad spectrum of sensations—spinning, imbalance, floating, or a sense of bodily disconnection—that are often difficult for patients to describe. Approximately 45% of patients with vertigo are seen by general practitioners (GPs) [2]. Vertigo also accounts for 4% of emergency department visits and is the most common presenting symptom of posterior circulation strokes, which represent around 20% of all ischemic strokes [3]. In this context, clinical examination is crucial to differentiate peripheral from central causes.

Acute vertigo management requires specific knowledge and clinical skills in otoneurology, which are not systematically included in GP training. In France, for instance, vestibular disorders are addressed during undergraduate medical education, with learning objectives set by the French College of Otorhinolaryngology [4]. Medical students in their final year are introduced to the theoretical foundations of vestibular syndrome assessment, with an emphasis on identifying red flags suggestive of central causes. However, the limited hands-on experience and clinical exposure at this stage may be insufficient for confidently managing patients with vertigo in real-world practice. This raises the question of how diagnostic reasoning in acute vertigo evolves between the end of undergraduate training and routine general practice, balancing recent theoretical knowledge and accumulated clinical experience.

Several validated tools have been developed to assess diagnostic reasoning in clinical settings using vignette-based assessments. Script Concordance Tests (SCTs) evaluate clinical decision-making under uncertainty and can differentiate levels of expertise  [5]. Key Feature Problems (KFPs) focus on essential decision points in clinical cases and have shown strong validity and reliability in assessing diagnostic reasoning [6].

Existing research on vertigo diagnosis has focused on emergency care settings, with limited data available from general practice [7]. To address this gap, we conducted a study comparing the diagnostic reasoning of practicing general practitioners and final-year medical students in France, using a standardized questionnaire based on complex cases of acute vertigo.

We hypothesized that diagnostic reasoning in the evaluation of acute vertigo would differ between general practitioners and final-year medical students, reflecting the balance between recent academic preparation and accumulated clinical experience.

## Materials and methods

### Ethics approval

This study consisted of a voluntary, anonymous survey of medical students and GPs. Prior to participation, all participants received an information notice describing the study objectives, procedures, and use of the collected data for research and publication purposes. All participants provided implied informed consent through voluntary, uncompensated participation. No patient data or clinical interventions were involved. The study protocol received approval from the Research Ethics Committee of the University Hospital of Rouen (approval No. E2025-58). We confirm that the entire questionnaire used in this study was fully developed by the authors and is made openly available under a CC BY 4.0 license.

### Study design and population

A multicenter, cross-sectional observational study was conducted via a self-administered online questionnaire, available from January to March 2024. The questionnaire focused on diagnostic reasoning assessed through vignette-based cases of acute vertigo. Participation was open to GPs practicing in Eastern Normandy and to final-year undergraduate medical students from the region’s medical school. The survey was distributed through the LimeSurvey© platform (LimeSurvey GmbH, Hamburg, Germany; URL: http://www.limesurvey.org).

According to data from the regional Medical Council, approximately 1,616 general practitioners were practicing in Eastern Normandy, including fully licensed physicians, locum doctors, collaborators, and residents. The questionnaire was distributed via email with the support of the Council and, simultaneously, it was sent to all final-year medical students enrolled at the University of Rouen. Incomplete questionnaires were excluded from the analysis. All participants provided implied informed consent through voluntary, uncompensated participation. Given the voluntary nature of participation, the risk of selection bias cannot be excluded.

### Questionnaire design

The questionnaire was developed and validated by three senior otorhinolaryngologists (S.D., F.C., C.M.) experienced in managing vestibular disorders and actively involved in teaching at the regional university hospital. It consisted of three parts: (i) practice-related information, (ii) a case-based diagnostic test, and (iii) self-perceived confidence in diagnosing acute vertigo. The full version of the questionnaire was intended for GPs, while medical students received only the case-based diagnostic test component.

GPs were asked to report their gender and years in clinical practice. Additional variables included practice setting (solo, multidisciplinary, or locum), average number of daily consultations, and weekly frequency of vertigo-related visits. They also indicated whether they had completed an ENT clinical rotation and whether they had participated in any vertigo-specific continuing medical education, such as theoretical courses, practical workshops or clinical simulations.

The case-based diagnostic test included five SCTs, each based on a distinct clinical vignette related to acute vertigo and composed of three diagnostic reasoning items. For each item, a diagnostic hypothesis was followed by a new clinical finding, and participants were asked to indicate whether the finding supported, weakened, or had no effect on the hypothesis, using a Likert scale. To establish the SCTs scoring key, responses were collected from a reference panel of 20 otorhinolaryngologists with varying levels of expertise, including residents, hospital-based physicians, and professors. Maximum points were awarded when a participant’s answer matched the majority response of the expert panel. In addition, three independent clinical scenarios were presented as KFPs. The same expert panel completed the KFPs and achieved a mean score of 84% (± 16%), with a normal distribution, supporting the overall quality and discriminatory capacity of the KFP items. Item-level scores from the SCTs and KFPs were summed to generate a total diagnostic reasoning score, with equal weighting of both components. All test items were developed in accordance with established guidelines, and their content was aligned with the learning objectives of the French medical curriculum program  [5,8].

A final item assessed participants’ confidence in managing acute vertigo using a binary response (yes/no). The full questionnaire and the information notice are available in Supporting Information (S1 and S2 File).

### Statistical analysis

Continuous variables were expressed as means (± standard deviation) or medians (interquartile range), and categorical variables as counts (percentages). Group comparisons used Student’s t-test or Mann–Whitney U test for continuous variables, and chi-square or Fisher’s exact test for categorical variables. To identify factors associated with higher diagnostic scores, univariate analyses were first conducted for the following characteristics: gender, training in the practice region, practice setting (solo vs. group), years in practice (≤10 vs. > 10), number of daily consultations (≤30 vs. > 30), prior ENT rotation, participation in vertigo-related training, frequency of vertigo consultations (≤5 vs. > 5/week), and self-reported confidence in vertigo management. A multivariable linear regression model was used to assess diagnostic reasoning as a continuous outcome, given the robustness of this approach to mild departures from normality. All tests were two-tailed, with p < 0.05 considered statistically significant. Analyses were performed using SPSS software (version 29.0.2.0; IBM Corp).

## Results

### Participant characteristics

A total of 265 participants fully completed the questionnaire between January and March 2024, including 173 general practitioners (GPs; 65.3%) and 92 final-year medical students (34.7%). Initially, 232 responses were received from GPs, of which 59 incomplete questionnaires were excluded from the analysis. The sample had a slight female majority (56.1%), with most participants practicing in group settings (89.6%) and approximately half having ≤10 years of clinical experience (50.9%). Approximately 60% had completed their undergraduate medical training in the region of practice, while 25% had undertaken vertigo-specific continuing medical education. The demographic and professional characteristics of the GPs are summarized in Table 1.

**Table 1:** Characteristics of respondent General Practitioners (n = 173).

Characteristic		n (%)
**Demographic Characteristics**		
Gender	Male	76 (43.9%)
	Female	97 (56.1%)
Type of practice setting	Solo practice	18 (10.4%)
	Group practice	155 (89.6%)
**Education and Training**		
Years of clinical experience	≤10 years	88 (50.9%)
	>10 years	85 (49.1%)
**Current Clinical Activity**		
Undergraduate training in the region of practice	Yes	103 (59.5%)
	No	70 (40.5%)
Completed ENT clinical rotation	Yes	29 (16.8%)
	No	144 (83.2%)
Completed vertigo-specific continuing medical education	Yes	44 (25.4%)
	No	129 (74.6%)
Average number of daily consultations	≤30	138 (79.8%)
	>30	35 (20.2%)
Average weekly vertigo-related consultations	≤5	141 (81.5%)
	>5	32 (18.5%)
Self-reported confidence in managing acute vertigo	Yes	25 (14.4%)
	No	148 (85.6%)

### Diagnostic reasoning and associated factors

Final-year medical students achieved higher scores than general practitioners on the vignette-based assessment of diagnostic reasoning in acute vertigo. The mean total score was significantly higher among students than among GPs (61.0% ± 12.9 vs. 54.0% ± 10.9; p < 0.001). This difference was observed across both assessment components, including the SCTs (58.7% vs. 53.1%; p = 0.003) and the KFPs (65.0% vs. 55.8%; p < 0.001) (Table 2). Although statistically significant, the absolute difference in mean scores was modest. The anonymized dataset supporting the findings of this study is available in Supporting Information (S3 Table).

**Table 2:** Results of the case-based diagnostic test for acute vertigo management.

Test component	GPs (n = 173) mean (%)	Final-year medical students (n = 92) mean (%)	p-value
Total score	54.0%	61.0%	<0.001*
Script Concordance Tests (SCT)	53.1%	58.7%	0.003*
Key Feature Problems (KFP)	55.8%	65.0%	<0.001*

* p-values indicate statistically significant differences. GP, general practitioner

Among GPs, univariate subgroup analyses identified several factors associated with higher diagnostic scores (Table 3). Statistically significant differences were found for daily consultation volume (≤30 patients: 55.0% vs. > 30 patients: 50.5%, p = 0.031), participation in vertigo-specific continuing medical education (57.0% vs. 53.0%, p = 0.047), and self-reported confidence in managing acute vertigo (60.0% vs. 53.0%, p = 0.003). Completion of an ENT rotation and frequency of vertigo consultations were not significantly associated with scores.

**Table 3:** Mean Diagnostic Test Scores (%) according to General Practitioner Characteristics (Univariate Analysis).

Characteristic	Category	n (%)	Mean score (%)	p-value
Gender	Male	76 (43.9%)	54.0%	0.953
	Female	97 (56.1%)	54.0%	
Undergraduate training in region of practice	Yes	103 (59.5%)	54.5%	0.474
	No	70 (40.5%)	53.5%	
Practice setting	Solo practice	18 (10.4%)	49.9%	0.079
	Group practice	155 (89.6%)	54.5%	
Years in clinical practice	≤ 10 years	88 (50.9%)	55.5%	0.084
	> 10 years	85 (49.1%)	52.5%	
Average daily consultations	≤ 30	138 (79.8%)	55.0%	0.031*
	> 30	35 (20.2%)	50.5%	
Completed ENT rotation	Yes	29 (16.8%)	53.0%	0.526
	No	144 (83.2%)	54.5%	
Completed vertigo-specific continuing medical education	Yes	44 (25.4%)	57.0%	0.047*
	No	129 (74.6%)	53.0%	
Weekly vertigo consultations	≤ 5	141 (81.5%)	54.5%	0.192
	> 5	32 (18.5%)	52.0%	
Self-reported diagnostic confidence	Yes	25 (14.4%)	60.0%	0.003*
	No	148 (85.6%)	53.0%	

* Statistically significant (p < 0.05).

In multivariable linear regression analysis, participation in vertigo-specific continuing medical education (β = 0.769, 95% CI [0.032; 1.506], p = 0.041) and self-reported diagnostic confidence (β = 1.449, 95% CI [0.542; 2.355], p = 0.002) remained independent predictors of higher scores, while other factors did not retain statistical significance (Table 4).

**Table 4:** Multivariable linear regression analysis of factors associated with higher diagnostic test scores among general practitioners.

Characteristic	Reference group	Regression coefficient (β)	95% Confidence Interval	p-value
Gender	Male	0.046	[-0.615; 0.707]	0.892
Undergraduate training in region of practice	Yes	0.249	[-0.404; 0.902]	0.453
Practice setting	Group practice	0.716	[-0.338; 1.769]	0.182
Years in clinical practice	> 10 years	−0.561	[-1.214; 0.092]	0.092
Average daily consultations	> 30	−0.795	[-1.661; 0.072]	0.072
Completed ENT rotation	Yes	−0.324	[-1.181; 0.534]	0.457
Completed vertigo-specific continuing medical education	Yes	0.769	[0.032; 1.506]	0.041*
Weekly vertigo consultations	> 5	−0.219	[-1.065; 0.627]	0.610
Self-reported diagnostic confidence	Yes	1.449	[0.542; 2.355]	0.002*

* Statistically significant (p < 0.05).

## Discussion

This study found that final-year medical students achieved higher scores on vignette-based assessments of diagnostic reasoning in acute vertigo than participating GPs from the same region. While it is unclear whether clinical experience or recent academic training would have a greater influence on diagnostic reasoning, the results suggest an association between proximity to medical training and higher diagnostic reasoning scores. Although the “school-like” format of the assessment may have favored students, these findings highlight the potential relevance of ongoing postgraduate training, particularly given that theoretical knowledge has been shown to decline after graduation [9,10]. SCTs have been used since 1998 to assess clinical reasoning in complex and uncertain cases across multiple disciplines, and have demonstrated their value in evaluating diagnostic reasoning skills [11–13]. The integration of SCTs and practical vestibular examination training into postgraduate education may represent a relevant educational approach for GPs.

GPs who reported participation in post-graduate training focused on vertigo achieved higher scores than their peers, suggesting an association between engagement in such programs and performance on vignette-based assessments. In France, GPs have been required to update their knowledge through post-graduate training since 2016; however, the content and availability of these programs vary across regions. To date, few studies have evaluated the effectiveness of these training programs. A 2022 Irish study reported that 74% of general practitioners perceived an improvement in patient care following participation in post-graduate training [14].

Self-reported confidence in diagnosing acute vertigo was independently associated with higher diagnostic reasoning scores among GPs. Although the exact role of this self-confidence is not fully understood, it may be related to prior training experiences. Confidence may influence how clinicians engage with diagnostic tasks and apply clinical reasoning strategies. However, confidence does not necessarily equate to competence; previous studies have reported that overconfidence may increase the risk of diagnostic errors, whereas low confidence may lead to unnecessary referrals or investigations [15]. From an educational perspective, postgraduate training should therefore aim not only to reinforce knowledge, but also to support the development of appropriately calibrated confidence.

Univariate analysis also indicated that GPs reporting fewer daily patients performed better at the tests. This association may reflect greater time availability per consultation or increased cognitive resources for reflective clinical reasoning. The clinical evaluation of patients presenting with acute vertigo can be time-consuming, particularly in the presence of nausea, vomiting, or anxiety. Longer consultation times may be associated with higher diagnostic reasoning and may also enhance GPs’ self-confidence and facilitate self-directed learning in vertigo diagnosis. However, consultation durations in primary care are often constrained and may not adequately reflect the healthcare needs of the population, with significant regional variability [16].

This study had several limitations. Final-year medical students may have been more familiar with structured assessment formats such as SCTs and KFPs, potentially conferring an advantage in this setting, although clear instructions were provided and the expert panel included otorhinolaryngologists unfamiliar with SCT methodology. The response rate among GPs was low (approximately 10%), which may have introduced selection bias, although participating GPs did not necessarily achieve higher diagnostic reasoning scores. In addition, it was not possible to verify whether participants consulted external resources while completing the questionnaire, although the information note encouraged spontaneous responses and the vignettes were designed to be complex and original. Medical students completed only the diagnostic reasoning component of the questionnaire, whereas general practitioners completed the full questionnaire, which may have influenced engagement or fatigue and should be considered when interpreting between-group comparisons. Participants’ self-reported confidence was assessed using a binary response (yes/no), which may oversimplify individual perceptions of confidence and does not capture its gradations; this limitation should be considered when interpreting associations between confidence and diagnostic reasoning.

Importantly, the findings of this study relate to diagnostic reasoning assessed through vignette-based tools and should not be interpreted as reflecting real-life clinical performance or actual patient management.

## Conclusion

**Fig 1:**
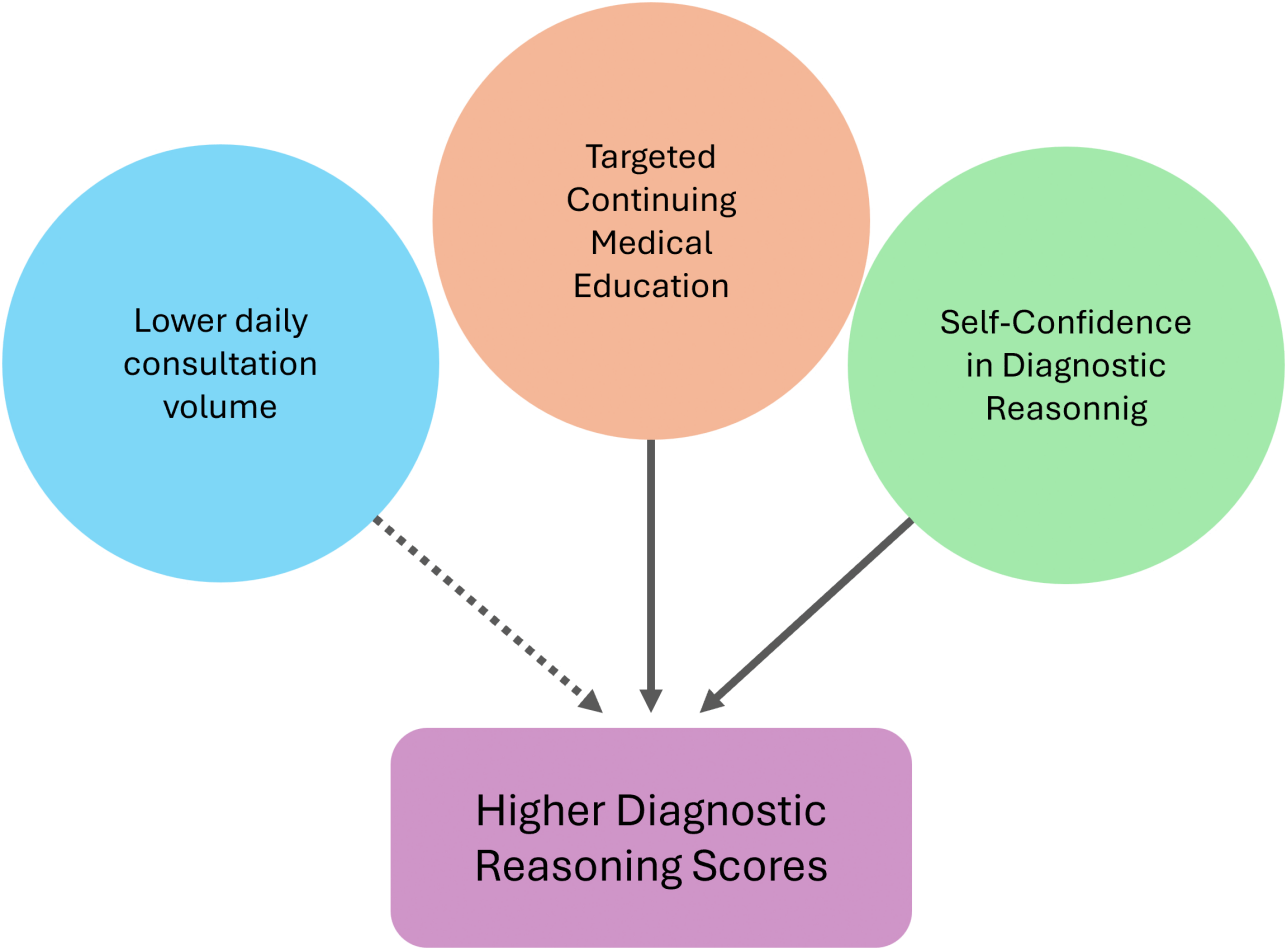
Factors associated with diagnostic reasoning in vignette-based assessments of acute vertigo among general practitioners. Solid arrows indicate factors independently associated with higher diagnostic reasoning scores in multivariable analysis, while dashed arrows indicate associations observed in univariate analyses only.

In conclusion, Fig 1 summarizes factors associated with higher diagnostic reasoning among general practitioners in vignette-based assessments of acute vertigo. These findings suggest that targeted continuing medical education may be associated with higher diagnostic reasoning in this context. Future educational approaches may benefit from integrating clinical cases inspired by real-life situations, combining theoretical input with practical application, particularly through patient simulations. These findings should be interpreted within the context of vignette-based assessments and do not directly reflect real-life clinical care.

## Supporting information

S1 File Questionnaire used for the assessment of diagnostic reasoning in acute vertigo. Full questionnaire used in the study, including participant demographics, prior training and clinical exposure to vertigo, Script Concordance Test (SCT) and Key Features Problems (KFP)–based clinical vignettes assessing diagnostic reasoning in acute vertigo.
(DOCX)

S2 File Participant information and consent statement. Information notice provided to participants prior to questionnaire completion, describing the study objectives, voluntary participation, anonymization of data, and consent for use of responses for research and publication purposes.
(DOCX)

S3 Table Anonymized study dataset. Fully anonymized dataset used for all analyses presented in the manuscript. The dataset contains no directly or indirectly identifying information.
(XLSX)
