# Supplementary material for: Assessment of diagnostic reasoning in acute vertigo using vignette-based tools: A cross-sectional comparison between general practitioners and final-year medical students
Source: PLoS One. 2026 Jul 15;21(7):e0347129. doi: 10.1371/journal.pone.0347129 (PMC13372113; doi:10.1371/journal.pone.0347129)
Supplement: S1 File — Full questionnaire used in the study, including participant demographics, prior training and clinical exposure to vertigo, Script Concordance Test (SCT) and Key Features Problems (KFP)–based clinical vignettes assessing diagnostic reasoning in acute vertigo. (DOCX) [file pone.0347129.s001.docx]

# S1 Appendix

**Appendix 1 – Questionnaire**

# Demographics

# What is your gender?

# ☐ Male

# ☐ Female

# What type of practice setting do you currently work in?

# ☐ Solo practice

# ☐ Group practice

# How many years of clinical experience do you have?

# ☐ ≤10 years

# ☐ >10 years

# Did you complete your undergraduate medical training in the region where you currently practice?

# ☐ Yes

# ☐ No

# Did you complete a clinical rotation in otorhinolaryngology (ENT) during your medical training?

# ☐ Yes

# ☐ No

# Have you completed any vertigo-specific continuing medical education?

# ☐ Yes

# ☐ No

# On average, how many consultations do you conduct per day?

# ☐ ≤30 consultations

# ☐ >30 consultations

# On average, how many patients do you see per week with vertigo as the presenting complaint?

# ☐ ≤5 patients

# ☐ >5 patients

# Participants’ confidence in managing acute vertigo diagnosis

1. Do you feel confident managing patients presenting with vertigo?
   ☐ Yes
   ☐ No

# II. SCT and KFP Diagnostic Assessment

**1)** You examine a 55-year-old patient with severe rotational vertigo and vomiting for 2 hours.

| **If you suspect** | **And you find** | **Effect on your diagnostic hypothesis (-2 = weakens strongly, 0 = no effect, +2 = strengthens strongly)** |
| --- | --- | --- |
| Posterior circulation stroke | Vertical nystagmus | -2 -1 0 +1 +2 |
| Central lesion | Bilateral tinnitus | -2 -1 0 +1 +2 |
| Vestibular neuritis | Rightward Romberg deviation and left nystagmus | -2 -1 0 +1 +2 |

**2)** You examine a 72-year-old woman with regular balance loss and recent acute worsening.

| **If you suspect** | **And you find** | **Effect on your diagnostic hypothesis (-2 to +2)** |
| --- | --- | --- |
| Ménière’s disease | Unilateral tinnitus | -2 -1 0 +1 +2 |
| Benign paroxysmal positional vertigo | Type 2 diabetes | -2 -1 0 +1 +2 |
| Phobic postural vertigo | Fall on Romberg test | -2 -1 0 +1 +2 |

**3)** You examine an 18-year-old patient with acute vertigo.

| **If you suspect** | **And you find** | **Effect on your diagnostic hypothesis (-2 to +2)** |
| --- | --- | --- |
| Vertebral artery dissection | Absence of nystagmus | -2 -1 0 +1 +2 |
| Acute labyrinthitis | Acute otitis media | -2 -1 0 +1 +2 |
| Vestibular migraine | History of otitis | -2 -1 0 +1 +2 |

**4)** You examine a 60-year-old patient presenting with sudden onset of unsteadiness and nausea lasting 4 hours.

| **If you suspect** | **And you find** | **Effect on your diagnostic hypothesis (-2 to +2)** |
| --- | --- | --- |
| Vestibular migraine | No hearing loss | -2 -1 0 +1 +2 |
| Brainstem stroke | Horizontal gaze-evoked nystagmus | -2 -1 0 +1 +2 |
| Labyrinthitis | Recent upper respiratory infection | -2 -1 0 +1 +2 |

**5)** You examine a 55-year-old patient with severe vertigo.

| **If you suspect** | **And you find** | **Effect on your diagnostic hypothesis (-2 to +2)** |
| --- | --- | --- |
| Central vestibular syndrome | Pathological test of skew | -2 -1 0 +1 +2 |
| Vestibular neuritis | Anxiety | -2 -1 0 +1 +2 |
| Stroke | Catch-up saccade on Halmagyi (Head Impulse) test | -2 -1 0 +1 +2 |

**5)** A 56-year-old patient presents with sudden severe rotational vertigo lasting 3 hours, vomiting, inability to stand, and eye closure.

Select **three** life-threatening emergencies to rule out urgently that could cause this presentation:

☐ Carotid artery dissection
☐ Left Sylvian ischemic stroke
☐ Cerebellar stroke
☐ Wallenberg syndrome
☐ Vertebral artery dissection
☐ Pyramidal syndrome
☐ Cavernous sinus thrombosis
☐ Sphenoidal sinusitis
☐ Parietal aneurysm rupture
☐ Bacterial meningitis
☐ Extrapyramidal syndrome

**6)** Among the following, which findings suggest a central cause?

☐ Abnormal finger-to-nose test
☐ Enlarged base of support (stance)
☐ Lateralized Romberg test
☐ Lateralized Fukuda test
☐ Unilateral hearing loss
☐ Abnormal otoscopy
☐ Presence of skew deviation
☐ Normal Halmagyi test
☐ Multidirectional nystagmus
☐ Unilateral tinnitus
☐ Tobacco use (10 cigarettes/day)
☐ Personal history of vertigo
☐ Family history of vertigo

**7)** The patient has a right peripheral vestibular syndrome. Which clinical elements support this diagnosis?

☐ Fever
☐ Anisocoria
☐ Facial paralysis
☐ Dysmetria
☐ Abnormal otoscopy
☐ History of similar vertigo episodes
☐ Swallowing difficulties
☐ Recent viral infection
☐ Non-febrile torticollis
