# Supplementary material for: Assessment of diagnostic reasoning in acute vertigo using vignette-based tools: A cross-sectional comparison between general practitioners and final-year medical students
Source: PLoS One. 2026 Jul 15;21(7):e0347129. doi: 10.1371/journal.pone.0347129 (PMC13372113; doi:10.1371/journal.pone.0347129)
Supplement: S2 File — Information notice provided to participants prior to questionnaire completion, describing the study objectives, voluntary participation, anonymization of data, and consent for use of responses for research and publication purposes. (DOCX) [file pone.0347129.s002.docx]

Information Notice for Participants

You are invited to take part in a research study evaluating clinical reasoning in the diagnosis and management of acute vertigo using written clinical vignettes.

Participation in this study is entirely voluntary. The questionnaire is anonymous, and no information allowing personal identification is collected. Your responses will be analyzed in aggregated form for research purposes only.

The data collected may be used for scientific analysis and publication in peer-reviewed journals. No individual data will be identifiable in any publication resulting from this study.

By completing the questionnaire, you confirm that you have read and understood this information and consent to the use of your anonymized data for research and publication purposes.

You may choose not to participate or to discontinue participation at any time without any consequences.
