# Supplementary material for: Assessment of diagnostic reasoning in acute vertigo using vignette-based tools: A cross-sectional comparison between general practitioners and final-year medical students
Source: PLoS One. 2026 Jul 15;21(7):e0347129. doi: 10.1371/journal.pone.0347129 (PMC13372113; doi:10.1371/journal.pone.0347129)
Supplement: Attachment — Submitted filename: Response to Reviewers.docx [file pone.0347129.s005.docx]

Response to the Editor – Journal Requirements

We thank the Editor and the Reviewers for their careful evaluation of our manuscript and for their constructive comments. We have revised the manuscript accordingly and believe that these changes have substantially improved its clarity, methodological transparency, and interpretability. Detailed responses are provided below.

1. PLOS ONE style requirements

The manuscript has been revised to comply with PLOS ONE style requirements, including formatting and file naming, in accordance with the provided PLOS ONE templates.

2. Funding statement and acknowledgments

All funding-related texts have been removed from the manuscript, including from the Acknowledgments section.

The Funding Statement remains unchanged and reads as follows, no modification to the Funding Statement is requested :

“The author(s) received no specific funding for this work.”

3. Ethics statement location

The ethics statement has been moved to and is now included exclusively in the Methods section of the manuscript. It has been removed from any other section, in accordance with PLOS ONE requirements.

4. Consent for publication and participant confidentiality

All participants provided informed consent to participate in the study and to the use of their data for research and publication purposes. Participants were informed at inclusion through an information notice, the existence of which has now been explicitly described in the manuscript.

The dataset does not contain any potentially identifying information. If required, the information notice can also be provided as Supporting Information.

5. Supporting Information captions

Captions for all Supporting Information files have been added at the end of the manuscript, and all in-text citations have been updated accordingly.

Response to Reviewers

## General Evaluation Questions

1. Technical soundness and support of conclusions

Reviewer #1: Partly

Response:

We acknowledge Reviewer #1’s concern regarding the initial overextension of conclusions. In response, we have carefully revised the manuscript to ensure that all conclusions are strictly supported by the data. The study has been consistently reframed as an educational assessment of diagnostic reasoning using vignette-based tools, rather than an evaluation of clinical diagnostic accuracy. Interpretive and causal language has been systematically removed or replaced with associative terminology throughout the manuscript, including the abstract, results, discussion, and conclusion.

2. Statistical analysis

Reviewer #1: No

Response:

The statistical analyses were not modified, but their rationale and presentation have been clarified. In particular:

- We clarified the aggregation of SCT and KFP scores into a single diagnostic reasoning score.
- We explicitly stated that SCT and KFP components were equally weighted.
- We added a brief justification for the use of linear regression to model diagnostic reasoning scores as a continuous outcome, noting the robustness of this approach.
- These clarifications were added to the Statistical Analysis section to improve transparency and reproducibility.

## Reviewer #1 – Specific Comments

Misclassification as “diagnostic accuracy”

Recommendation:

Reframe the study consistently as an educational assessment of diagnostic reasoning, not a diagnostic accuracy study. The title, abstract, and conclusions should be modified accordingly.

Response:

- We fully agree with this comment. The manuscript has been comprehensively revised to:
- Replace all instances of diagnostic accuracy with diagnostic reasoning.
- Explicitly state that SCTs and KFPs assess diagnostic reasoning under uncertainty using vignette-based tools.
- Reframe the title, abstract, results, discussion, figure legends, and conclusion accordingly.
- Additionally, we now explicitly state in the Discussion that the findings do not reflect real-life clinical performance or patient management.

## Reviewer #2 – Section-by-Section Responses

### Abstract

Issues & Recommendations:

Interpretive language should be slightly tempered

The conclusion attributes the superior performance of students mainly to ‘’ recent targeted training. ‘’While plausible, this inference is not directly tested.

Recommend softening to:

‘’…possibly reflecting more recent theoretical exposure.’’

Clarify clinical vs. Test-based performance

Readers may interpret ‘’ diagnostic accuracy’’ as real-clinical accuracy

Suggest adding ‘’ in vignette-based assessments’’ to the conclusion sentence.

Response :

- Interpretive language was tempered.
- References to “recent targeted training” were softened to possibly reflecting more recent theoretical exposure.
- The conclusion now explicitly refers to vignette-based assessments of diagnostic reasoning.

### Introduction

1. Provide clarification of the educational paradox earlier in the introduction.

- The statement on the unexpected hypothesis (students have superior diagnostic skills to GPs) appears relatively late in the introduction.

2. As a way of clarifying what the authors mean by "educational paradox," the authors may want to include the following statement in the final paragraph of the introduction: "This study looks at the balance between a student's academic preparation and the clinical experience of a GP when conducting structured clinical tests (SCTs)."

Responses :

1. The “educational paradox” is introduced earlier in the Introduction.
2. The final paragraph now explicitly frames the study as examining the balance between recent academic preparation and accumulated clinical experience when completing structured clinical tests (SCTs and KFPs).

### 3. Materials and Methods

3.1 Study Design and Population

- Response rate bias was an issue.

- The physician response rate (approximately 10%) is discussed later in the paper; it should have been addressed earlier in the paper.

- Include a brief statement addressing the potential for selection bias.

- There is an imbalance between how many times students and doctors were exposed to this study's content.

- Students only participated in completing the diagnostic section of this study.

- Clarify if this difference in exposure may lead to differences in levels of fatigue or engagement when participating.

Response :

The low response rate among general practitioners (~10%) is now reported in the Methods.

A statement acknowledging potential selection bias has been added.

Differences in questionnaire exposure between students and GPs are clearly described.

3.2 Questionnaire Design

Explain how the scores were aggregated to obtain the total score.

Clarify if the SCT and KFP scores are equally weighted in the total score.

This would provide more transparency and reproducibility.

Confidence Assessment

The confidence question is binary (yes/no), which may oversimplify the perception of the participant. This limitation should be explicitly mentioned.

Response:

The method used to aggregate SCT and KFP scores into a total diagnostic reasoning score is explicitly described.

Equal weighting of SCT and KFP components is stated.

The binary nature of the confidence question (yes/no) is specified.

3.3 Statistical Analysis

Briefly describe why linear regression was used as the analysis method. (normality of residuals or robustness of the regression analysis).

Response:

A justification for the use of linear regression has been added, highlighting its appropriateness and robustness.

### 4. Results

Clinical vs. Statistical Significance.

The absolute difference in percentages between groups (~7%).

Add a single sentence explaining whether this statistic can be considered educationally meaningful.

Do not over-analyze or conclude.

The written results section is primarily objective; thus, it should not include any type of causal terminology.

Response:

The Results section now strictly uses descriptive, non-causal language.

A single sentence clarifies that, although statistically significant, the absolute difference in mean scores (~7%) was modest, without interpreting its educational relevance.

### 5. Discussion

Comments addressed: Assessment Format Bias

In the discussion, students may be advantaged by school-type assessments.

Suggest further expansion on the limits of Construct Validity.

Confidence vs. Competence

Discussion of the confidence-calibration concept is well thought out.

Suggest reiterating that confidence is an educational target and not the endpoint of education.

Figure 1 Interpretation

The legend for Figure 1 describes the figure's findings using interpretative terminology (ie, "appears to promote better management").

Suggest using terminology consistent with PLOS ONE to label this finding, for example, "Higher Diagnostic Scores are associated with these Factors."

Response:

Potential bias related to the vignette-based assessment format is explicitly discussed.

The limits of construct validity are expanded.

The distinction between confidence and competence is reinforced, emphasizing confidence as an educational target rather than an endpoint.

Figure 1 legend has been revised to use strictly associative terminology (associated with).

A clear statement now specifies that findings relate to diagnostic reasoning assessed through vignette-based tools and should not be interpreted as reflecting real-life clinical care.

### 6. Conclusion

Comments addressed: In regard to recommendations, there will be recommendations to replace residual phrases regarding causation with phrases of association and to clarify that these findings relate to performance on diagnostic reasoning, not performance on clinical care.

Response: Residual causal language was removed.

The conclusion now explicitly states that findings pertain to vignette-based diagnostic reasoning and not to clinical care outcomes.

7. Ethics, Data Availability, and Transparency

Response: Modifications done in editor section

### 8. Language and Presentation

Response:

Minor typographical and stylistic issues were corrected. The manuscript was carefully edited to ensure clarity and consistency.
