# Supplementary material for: Assessment of diagnostic reasoning in acute vertigo using vignette-based tools: A cross-sectional comparison between general practitioners and final-year medical students
Source: PLoS One. 2026 Jul 15;21(7):e0347129. doi: 10.1371/journal.pone.0347129 (PMC13372113; doi:10.1371/journal.pone.0347129)
Supplement: Peer review history

# Peer review: Decision Letter 0

**DOI:** 10.1371/journal.pone.0347129.r001

**Authors:** Renato Melo

30 Dec 2025

PONE-D-25-58696

Comparing Diagnostic Accuracy of Acute Vertigo Between General Practitioners and Medical Students: A Cross-Sectional Study

PLOS One

Dear Dr. Maquet,

Thank you for submitting your manuscript to PLOS ONE. After careful consideration, we feel that it has merit but does not fully meet PLOS ONE’s publication criteria as it currently stands. Therefore, we invite you to submit a revised version of the manuscript that addresses the points raised during the review process.

Please submit your revised manuscript by Feb 13 2026 11:59PM. If you will need more time than this to complete your revisions, please reply to this message or contact the journal office at plosone@plos.org. When you're ready to submit your revision, log on to https://www.editorialmanager.com/pone/ and select the 'Submissions Needing Revision' folder to locate your manuscript file.

Please include the following items when submitting your revised manuscript:

- A letter that responds to each point raised by the academic editor and reviewer(s). You should upload this letter as a separate file labeled 'Response to Reviewers'.
- A marked-up copy of your manuscript that highlights changes made to the original version. You should upload this as a separate file labeled 'Revised Manuscript with Track Changes'.
- An unmarked version of your revised paper without tracked changes. You should upload this as a separate file labeled 'Manuscript'.

If you would like to make changes to your financial disclosure, please include your updated statement in your cover letter. Guidelines for resubmitting your figure files are available below the reviewer comments at the end of this letter.

If applicable, we recommend that you deposit your laboratory protocols in protocols.io to enhance the reproducibility of your results. Protocols.io assigns your protocol its own identifier (DOI) so that it can be cited independently in the future. For instructions see: https://journals.plos.org/plosone/s/submission-guidelines#loc-laboratory-protocols. Additionally, PLOS ONE offers an option for publishing peer-reviewed Lab Protocol articles, which describe protocols hosted on protocols.io. Read more information on sharing protocols at https://plos.org/protocols?utm_medium=editorial-email&utm_source=authorletters&utm_campaign=protocols.

We look forward to receiving your revised manuscript.

Kind regards,

Renato S. Melo, PhD

Academic Editor

PLOS One

**Journal Requirements:**

1. When submitting your revision, we need you to address these additional requirements.

Please ensure that your manuscript meets PLOS ONE's style requirements, including those for file naming. The PLOS ONE style templates can be found at

https://journals.plos.org/plosone/s/file?id=wjVg/PLOSOne_formatting_sample_main_body.pdf and

https://journals.plos.org/plosone/s/file?id=ba62/PLOSOne_formatting_sample_title_authors_affiliations.pdf

2. Thank you for stating the following in the Acknowledgments Section of your manuscript:

The authors acknowledge the assistance of ChatGPT 4.0 in translation refinement. This study received no external funding. The authors have declared that no competing interests exist.

We note that you have provided funding information that is not currently declared in your Funding Statement. However, funding information should not appear in the Acknowledgments section or other areas of your manuscript. We will only publish funding information present in the Funding Statement section of the online submission form.

Please remove any funding-related text from the manuscript and let us know how you would like to update your Funding Statement. Currently, your Funding Statement reads as follows:

The author(s) received no specific funding for this work.

Please include your amended statements within your cover letter; we will change the online submission form on your behalf.

3. Your ethics statement should only appear in the Methods section of your manuscript. If your ethics statement is written in any section besides the Methods, please move it to the Methods section and delete it from any other section. Please ensure that your ethics statement is included in your manuscript, as the ethics statement entered into the online submission form will not be published alongside your manuscript.

4. We note that this data set consists of interview transcripts. Can you please confirm that all participants gave consent for interview transcript to be published?

If they DID provide consent for these transcripts to be published, please also confirm that the transcripts do not contain any potentially identifying information (or let us know if the participants consented to having their personal details published and made publicly available). We consider the following details to be identifying information:

- Names, nicknames, and initials

- Age more specific than round numbers

- GPS coordinates, physical addresses, IP addresses, email addresses

- Information in small sample sizes (e.g. 40 students from X class in X year at X university)

- Specific dates (e.g. visit dates, interview dates)

- ID numbers

Or, if the participants DID NOT provide consent for these transcripts to be published:

- Provide a de-identified version of the data or excerpts of interview responses

- Provide information regarding how these transcripts can be accessed by researchers who meet the criteria for access to confidential data, including:

a) the grounds for restriction

b) the name of the ethics committee, Institutional Review Board, or third-party organization that is imposing sharing restrictions on the data

c) a non-author, institutional point of contact that is able to field data access queries, in the interest of maintaining long-term data accessibility.

d) Any relevant data set names, URLs, DOIs, etc. that an independent researcher would need in order to request your minimal data set.

For further information on sharing data that contains sensitive participant information, please see: https://journals.plos.org/plosone/s/data-availability#loc-human-research-participant-data-and-other-sensitive-data

If there are ethical, legal, or third-party restrictions upon your dataset, you must provide all of the following details (https://journals.plos.org/plosone/s/data-availability#loc-acceptable-data-access-restrictions):

a) A complete description of the dataset

b) The nature of the restrictions upon the data (ethical, legal, or owned by a third party) and the reasoning behind them

c) The full name of the body imposing the restrictions upon your dataset (ethics committee, institution, data access committee, etc)

d) If the data are owned by a third party, confirmation of whether the authors received any special privileges in accessing the data that other researchers would not have

e) Direct, non-author contact information (preferably email) for the body imposing the restrictions upon the data, to which data access requests can be sent

5. Please include captions for your Supporting Information files at the end of your manuscript, and update any in-text citations to match accordingly. Please see our Supporting Information guidelines for more information: http://journals.plos.org/plosone/s/supporting-information.

6. If the reviewer comments include a recommendation to cite specific previously published works, please review and evaluate these publications to determine whether they are relevant and should be cited. There is no requirement to cite these works unless the editor has indicated otherwise.

[Note: HTML markup is below. Please do not edit.]

Reviewers' comments:

Reviewer's Responses to Questions

**Comments to the Author**

1. Is the manuscript technically sound, and do the data support the conclusions?

The manuscript must describe a technically sound piece of scientific research with data that supports the conclusions. Experiments must have been conducted rigorously, with appropriate controls, replication, and sample sizes. The conclusions must be drawn appropriately based on the data presented.

Reviewer #1: Partly

Reviewer #2: Yes

**********

2. Has the statistical analysis been performed appropriately and rigorously?

Reviewer #1: No

Reviewer #2: Yes

**********

3. Have the authors made all data underlying the findings in their manuscript fully available?

The PLOS Data policy requires authors to make all data underlying the findings described in their manuscript fully available without restriction, with rare exception (please refer to the Data Availability Statement in the manuscript PDF file). The data should be provided as part of the manuscript or its supporting information, or deposited to a public repository. For example, in addition to summary statistics, the data points behind means, medians and variance measures should be available. If there are restrictions on publicly sharing data—e.g. participant privacy or use of data from a third party—those must be specified.

Reviewer #1: Yes

Reviewer #2: Yes

**********

4. Is the manuscript presented in an intelligible fashion and written in standard English?

PLOS ONE does not copyedit accepted manuscripts, so the language in submitted articles must be clear, correct, and unambiguous. Any typographical or grammatical errors should be corrected at revision, so please note any specific errors here.

Reviewer #1: Yes

Reviewer #2: Yes

**********

5. Review Comments to the Author

Please use the space provided to explain your answers to the questions above. You may also include additional comments for the author, including concerns about dual publication, research ethics, or publication ethics. (Please upload your review as an attachment if it exceeds 20,000 characters)

**Reviewer #1:**  This manuscript reports a cross-sectional comparison of vignette-based diagnostic performance in acute vertigo between general practitioners (GPs) and final-year medical students, using Script Concordance Tests (SCTs) and Key Feature Problems (KFPs). The topic is relevant to primary care education, and the use of validated assessment tools is a strength. However, important methodological and interpretative limitations substantially weaken the clinical implications of the findings. In its current form, the manuscript overextends its conclusions beyond what the data can robustly support.1. Misclassification of the study as “diagnostic accuracy”

The manuscript repeatedly refers to “diagnostic accuracy,” yet no real patients, reference clinical diagnoses, or patient outcomes are included.The study assesses performance on educational assessment tools, not diagnostic accuracy in the clinical sense.SCTs and KFPs evaluate clinical reasoning under uncertainty, not bedside diagnostic competence.

Recommendation:

Reframe the study consistently as an educational assessment of diagnostic reasoning, not a diagnostic accuracy study. The title, abstract, and conclusions should be modified accordingly.

**Reviewer #2:**  This manuscript addresses an important and under-studied issue in primary care and medical education: diagnostic reasoning in acute vertigo, comparing general practitioners and final-year medical students using validated assessment tools. The study is methodologically sound, ethically conducted, and clearly reported, and the conclusions are, to a great extent, supported by the data.

The work is appropriate for publication by PLOS ONE as an observational study that has been rigorously conducted and has clearly reported its methodology. However, I believe that the use of some of the conceptual clarification, methodological refinement, and presentation issues will strengthen the interpretability, reproducibility, and balance of the conclusions drawn.

I have provided comments and actionable recommendations on a section-by-section basis.

1. Abstract

Strengths

• Clear structure with standard PLOS ONE headings.

• Objectives, methods, and main findings are accurately reported.

• Effects, estimates, and p-values are appropriately reported.

Issues & Recommendations

1. Interpretive language should be slightly tempered

o The conclusion attributes the superior performance of students mainly to ‘’ recent targeted training. ‘’While plausible, this inference is not directly tested.

o Recommend softening to:

o ‘’…possibly reflecting more recent theoretical exposure.’’

2. Clarify clinical vs. Test-based performance

o Readers may interpret ‘’ diagnostic accuracy’’ as real-clinical accuracy

o Suggest adding ‘’ in vignette-based assessments’’ to the conclusion sentence.

2. Introductory

Strengths

- The introduction contains an appropriate epidemiological context.

- The introduction states the importance of vertigo for primary care practitioners.

- The paper correctly identifies and names structured clinical tests (SCTs) and knowledge and feedback protocols (KFPs) using citation support.

Issues & Recommendations

1. Provide clarification of the educational paradox earlier in the introduction.

- The statement on the unexpected hypothesis (students have superior diagnostic skills to GPs) appears relatively late in the introduction.

2. As a way of clarifying what the authors mean by "educational paradox," the authors may want to include the following statement in the final paragraph of the introduction: "This study looks at the balance between a student's academic preparation and the clinical experience of a GP when conducting structured clinical tests (SCTs)."

3. Materials and Methods

3.1 Study Design and Population

Strengths

- The study had clearly defined eligibility criteria.

- The recruitment approach was transparent.

- There were appropriate ethical considerations in the project.

Issues and Recommendations

- Response rate bias was an issue.

- The physician response rate (approximately 10%) is discussed later in the paper; it should have been addressed earlier in the paper.

- Include a brief statement addressing the potential for selection bias.

- There is an imbalance between how many times students and doctors were exposed to this study's content.

- Students only participated in completing the diagnostic section of this study.

- Clarify if this difference in exposure may lead to differences in levels of fatigue or engagement when participating.

3.2 Design of the Questionnaire

Strengths

The questionnaire's use of validated tools (the SCT and KFP) is a significant strength. In addition, there is a good description of how the expert panel was developed, as well as the way in which the expert panel's scoring was carried out. There are also good supporting materials.

Issues & Recommendations

Explain how the scores were aggregated to obtain the total score.

Clarify if the SCT and KFP scores are equally weighted in the total score.

This would provide more transparency and reproducibility.

Confidence Assessment

The confidence question is binary (yes/no), which may oversimplify the perception of the participant. This limitation should be explicitly mentioned.

3.3 Statistical Analysis

Strengths

- Univariate and multivariable analysis methods were appropriate to the study's objectives.

- The regression model was reported, including reporting of coefficients and confidence intervals.

- The software used and the threshold for significance were reported.

Minor Recommendations

- Briefly describe why linear regression was used as the analysis method. (normality of residuals or robustness of the regression analysis).

4. Results

Strengths

Logical connection of client attributes to results from performance.

The tables provided are organized with clarity and are all labelled appropriately, informing the reader about their content.

The rates of effect size associated with the tables are consistently presented throughout the text.

Issues & Recommendations

Clinical vs. Statistical Significance.

The absolute difference in percentages between groups (~7%).

Add a single sentence explaining whether this statistic can be considered educationally meaningful.

Do not over-analyze or conclude.

The written results section is primarily objective; thus, it should not include any type of causal terminology.

5. Discussion

Strengths

Both systemic and educational Elements are equally accounted for.

Knowledge Decay and Continuing Education Resources were cited and included in the literature review.

Limitations were noted.

Issue & Recommendations

Assessment Format Bias

In the discussion, students may be advantaged by school-type assessments.

Suggest further expansion on the limits of Construct Validity.

Confidence vs. Competence

Discussion of the confidence-calibration concept is well thought out.

Suggest reiterating that confidence is an educational target and not the endpoint of education.

Figure 1 Interpretation

The legend for Figure 1 describes the figure's findings using interpretative terminology (ie, "appears to promote better management").

Suggest using terminology consistent with PLOS ONE to label this finding, for example, "Higher Diagnostic Scores are associated with these Factors."

6. Conclusion

Strengths

The findings of this report are concise and establish a solid connection to the findings.

A clear request was made for continued medical education.

Recommendation

In regard to recommendations, there will be recommendations to replace residual phrases regarding causation with phrases of association and to clarify that these findings relate to performance on diagnostic reasoning, not performance on clinical care.

7. Ethics, Data Availability, and Transparency

Minor Note: The ethics approval numbers appear to be somewhat inconsistent between these sections; please align them to maintain continuity.

8. Language and Presentation: English was written in a clear and professional tone without errors in grammar or style. A few minor typographical and stylistic corrections and adjustments, such as spacing, hyphenation, etc., have been suggested. I suggest that light editorial revisions prior to acceptance be made.

**********

6. PLOS authors have the option to publish the peer review history of their article (what does this mean?). If published, this will include your full peer review and any attached files.

If you choose “no”, your identity will remain anonymous but your review may still be made public.

**Do you want your identity to be public for this peer review?**  For information about this choice, including consent withdrawal, please see our Privacy Policy.

Reviewer #1: **Yes:**  Sergio Carmona

Reviewer #2: No

**********

[NOTE: If reviewer comments were submitted as an attachment file, they will be attached to this email and accessible via the submission site. Please log into your account, locate the manuscript record, and check for the action link "View Attachments". If this link does not appear, there are no attachment files.]

To ensure your figures meet our technical requirements, please review our figure guidelines: https://journals.plos.org/plosone/s/figures

You may also use PLOS’s free figure tool, NAAS, to help you prepare publication quality figures: https://journals.plos.org/plosone/s/figures#loc-tools-for-figure-preparation.

NAAS will assess whether your figures meet our technical requirements by comparing each figure against our figure specifications.

# Author comment: Author response to Decision Letter 1

**DOI:** 10.1371/journal.pone.0347129.r002

15 Jan 2026

General Evaluation Questions

1. Technical soundness and support of conclusions

Reviewer #1: Partly

Response:

We acknowledge Reviewer #1’s concern regarding the initial overextension of conclusions. In response, we have carefully revised the manuscript to ensure that all conclusions are strictly supported by the data. The study has been consistently reframed as an educational assessment of diagnostic reasoning using vignette-based tools, rather than an evaluation of clinical diagnostic accuracy. Interpretive and causal language has been systematically removed or replaced with associative terminology throughout the manuscript, including the abstract, results, discussion, and conclusion.

2. Statistical analysis

Reviewer #1: No

Response:

The statistical analyses were not modified, but their rationale and presentation have been clarified. In particular:

- We clarified the aggregation of SCT and KFP scores into a single diagnostic reasoning score.

- We explicitly stated that SCT and KFP components were equally weighted.

- We added a brief justification for the use of linear regression to model diagnostic reasoning scores as a continuous outcome, noting the robustness of this approach.

- These clarifications were added to the Statistical Analysis section to improve transparency and reproducibility.

Reviewer #1 – Specific Comments

Misclassification as “diagnostic accuracy”

Recommendation:

Reframe the study consistently as an educational assessment of diagnostic reasoning, not a diagnostic accuracy study. The title, abstract, and conclusions should be modified accordingly.

Response:

- We fully agree with this comment. The manuscript has been comprehensively revised to:

- Replace all instances of diagnostic accuracy with diagnostic reasoning.

- Explicitly state that SCTs and KFPs assess diagnostic reasoning under uncertainty using vignette-based tools.

- Reframe the title, abstract, results, discussion, figure legends, and conclusion accordingly.

- Additionally, we now explicitly state in the Discussion that the findings do not reflect real-life clinical performance or patient management.

Reviewer #2 – Section-by-Section Responses

Abstract

Issues & Recommendations:

Interpretive language should be slightly tempered

The conclusion attributes the superior performance of students mainly to ‘’ recent targeted training. ‘’While plausible, this inference is not directly tested.

Recommend softening to:

‘’…possibly reflecting more recent theoretical exposure.’’

Clarify clinical vs. Test-based performance

Readers may interpret ‘’ diagnostic accuracy’’ as real-clinical accuracy

Suggest adding ‘’ in vignette-based assessments’’ to the conclusion sentence.

Response :

- Interpretive language was tempered.

- References to “recent targeted training” were softened to possibly reflecting more recent theoretical exposure.

- The conclusion now explicitly refers to vignette-based assessments of diagnostic reasoning.

Introduction

1. Provide clarification of the educational paradox earlier in the introduction.

- The statement on the unexpected hypothesis (students have superior diagnostic skills to GPs) appears relatively late in the introduction.

2. As a way of clarifying what the authors mean by "educational paradox," the authors may want to include the following statement in the final paragraph of the introduction: "This study looks at the balance between a student's academic preparation and the clinical experience of a GP when conducting structured clinical tests (SCTs)."

Responses :

1. The “educational paradox” is introduced earlier in the Introduction.

2. The final paragraph now explicitly frames the study as examining the balance between recent academic preparation and accumulated clinical experience when completing structured clinical tests (SCTs and KFPs).

3. Materials and Methods

3.1 Study Design and Population

- Response rate bias was an issue.

- The physician response rate (approximately 10%) is discussed later in the paper; it should have been addressed earlier in the paper.

- Include a brief statement addressing the potential for selection bias.

- There is an imbalance between how many times students and doctors were exposed to this study's content.

- Students only participated in completing the diagnostic section of this study.

- Clarify if this difference in exposure may lead to differences in levels of fatigue or engagement when participating.

Response :

The low response rate among general practitioners (~10%) is now reported in the Methods.

A statement acknowledging potential selection bias has been added.

Differences in questionnaire exposure between students and GPs are clearly described.

3.2 Questionnaire Design

Explain how the scores were aggregated to obtain the total score.

Clarify if the SCT and KFP scores are equally weighted in the total score.

This would provide more transparency and reproducibility.

Confidence Assessment

The confidence question is binary (yes/no), which may oversimplify the perception of the participant. This limitation should be explicitly mentioned.

Response:

The method used to aggregate SCT and KFP scores into a total diagnostic reasoning score is explicitly described.

Equal weighting of SCT and KFP components is stated.

The binary nature of the confidence question (yes/no) is specified.

3.3 Statistical Analysis

Briefly describe why linear regression was used as the analysis method. (normality of residuals or robustness of the regression analysis).

Response:

A justification for the use of linear regression has been added, highlighting its appropriateness and robustness.

4. Results

Clinical vs. Statistical Significance.

The absolute difference in percentages between groups (~7%).

Add a single sentence explaining whether this statistic can be considered educationally meaningful.

Do not over-analyze or conclude.

The written results section is primarily objective; thus, it should not include any type of causal terminology.

Response:

The Results section now strictly uses descriptive, non-causal language.

A single sentence clarifies that, although statistically significant, the absolute difference in mean scores (~7%) was modest, without interpreting its educational relevance.

5. Discussion

Comments addressed: Assessment Format Bias

In the discussion, students may be advantaged by school-type assessments.

Suggest further expansion on the limits of Construct Validity.

Confidence vs. Competence

Discussion of the confidence-calibration concept is well thought out.

Suggest reiterating that confidence is an educational target and not the endpoint of education.

Figure 1 Interpretation

The legend for Figure 1 describes the figure's findings using interpretative terminology (ie, "appears to promote better management").

Suggest using terminology consistent with PLOS ONE to label this finding, for example, "Higher Diagnostic Scores are associated with these Factors."

Response:

Potential bias related to the vignette-based assessment format is explicitly discussed.

The limits of construct validity are expanded.

The distinction between confidence and competence is reinforced, emphasizing confidence as an educational target rather than an endpoint.

Figure 1 legend has been revised to use strictly associative terminology (associated with).

A clear statement now specifies that findings relate to diagnostic reasoning assessed through vignette-based tools and should not be interpreted as reflecting real-life clinical care.

6. Conclusion

Comments addressed: In regard to recommendations, there will be recommendations to replace residual phrases regarding causation with phrases of association and to clarify that these findings relate to performance on diagnostic reasoning, not performance on clinical care.

Response: Residual causal language was removed.

The conclusion now explicitly states that findings pertain to vignette-based diagnostic reasoning and not to clinical care outcomes.

7. Ethics, Data Availability, and Transparency

Response: Modifications done in editor section

8. Language and Presentation

Response:

Minor typographical and stylistic issues were corrected. The manuscript was carefully edited to ensure clarity and consistency.

Attachment Submitted filename: Response to Reviewers.docx

# Peer review: Decision Letter 1

**DOI:** 10.1371/journal.pone.0347129.r003

**Authors:** Renato Melo, Renato Melo

29 Mar 2026

Assessment of diagnostic reasoning in acute vertigo using vignette-based tools: a cross-sectional comparison between general practitioners and final-year medical students

PONE-D-25-58696R1

Dear Dr. Maquet,

We’re pleased to inform you that your manuscript has been judged scientifically suitable for publication and will be formally accepted for publication once it meets all outstanding technical requirements.

Within one week, you’ll receive an e-mail detailing the required amendments. When these have been addressed, you’ll receive a formal acceptance letter and your manuscript will be scheduled for publication.

An invoice will be generated when your article is formally accepted. Please note, if your institution has a publishing partnership with PLOS and your article meets the relevant criteria, all or part of your publication costs will be covered. Please make sure your user information is up-to-date by logging into Editorial Manager at Editorial Manager® and clicking the ‘Update My Information' link at the top of the page. For questions related to billing, please contact billing support.

If your institution or institutions have a press office, please notify them about your upcoming paper to help maximize its impact. If they’ll be preparing press materials, please inform our press team as soon as possible -- no later than 48 hours after receiving the formal acceptance. Your manuscript will remain under strict press embargo until 2 pm Eastern Time on the date of publication. For more information, please contact onepress@plos.org.

Kind regards,

Renato S. Melo, PhD

Academic Editor

PLOS One

Additional Editor Comments (optional):

Reviewers' comments:

Reviewer's Responses to Questions

**Comments to the Author**

1. If the authors have adequately addressed your comments raised in a previous round of review and you feel that this manuscript is now acceptable for publication, you may indicate that here to bypass the “Comments to the Author” section, enter your conflict of interest statement in the “Confidential to Editor” section, and submit your "Accept" recommendation.

Reviewer #1: All comments have been addressed

Reviewer #2: All comments have been addressed

**********

2. Is the manuscript technically sound, and do the data support the conclusions?

The manuscript must describe a technically sound piece of scientific research with data that supports the conclusions. Experiments must have been conducted rigorously, with appropriate controls, replication, and sample sizes. The conclusions must be drawn appropriately based on the data presented.

Reviewer #1: Yes

Reviewer #2: Yes

**********

3. Has the statistical analysis been performed appropriately and rigorously?

Reviewer #1: Yes

Reviewer #2: Yes

**********

4. Have the authors made all data underlying the findings in their manuscript fully available?

The PLOS Data policy requires authors to make all data underlying the findings described in their manuscript fully available without restriction, with rare exception (please refer to the Data Availability Statement in the manuscript PDF file). The data should be provided as part of the manuscript or its supporting information, or deposited to a public repository. For example, in addition to summary statistics, the data points behind means, medians and variance measures should be available. If there are restrictions on publicly sharing data—e.g. participant privacy or use of data from a third party—those must be specified.

Reviewer #1: Yes

Reviewer #2: (No Response)

**********

5. Is the manuscript presented in an intelligible fashion and written in standard English?

PLOS ONE does not copyedit accepted manuscripts, so the language in submitted articles must be clear, correct, and unambiguous. Any typographical or grammatical errors should be corrected at revision, so please note any specific errors here.

Reviewer #1: Yes

Reviewer #2: Yes

**********

6. Review Comments to the Author

Please use the space provided to explain your answers to the questions above. You may also include additional comments for the author, including concerns about dual publication, research ethics, or publication ethics. (Please upload your review as an attachment if it exceeds 20,000 characters)

Reviewer #1: The issue is not only that vignette-based tools do not reflect real-life performance, but that in acute vestibular syndrome, diagnostic reasoning is inseparable from bedside examination (e.g., oculomotor findings). Therefore, vignette-based assessments may lack construct validity for this specific clinical domain, limiting the interpretability of comparisons between learners and practitioners

Reviewer #2: I’d like to take a moment to thank you for the opportunity to comment on this revised version of the manuscript. I see that the authors have very effectively addressed many of the comments from the previous review cycle, especially those comments related to issues of interpretive overreach, construct validity, and clarifying the definition of what is and is not being measured.

Abstract and Title:

The authors have revised the abstract and title to more accurately reflect the vignette-based nature of the assessment of diagnostic reasoning and less on how accurately it can diagnose clinically. The author's interpretive language has been toned down, and the conclusions drawn in the study now reflect the design of the study.

Introduction:

The authors nicely frame the educational context by explaining that an “educational paradox” exists when there is more emphasis placed on theoretical content versus what has been gained through experience. The hypothesis of the study is presented in a more appropriate position and provides a more cohesive rationale for the decision to use SCTs and KFPs.

Methods:

I can now see that the authors have provided detailed descriptions of the study population, how the questionnaires were constructed, and how the Expert Panel was created. By including detailed explanations of how the scores were aggregated and equally weighed between the SCT and KFP scores, the authors have increased the amount of transparency and reproducibility of the methods. The authors appropriately acknowledge that selection bias may have occurred as a result of response rates or different levels of exposure to the subjects.

Statistical Analysis:

The statistical methods employed by the authors appear to be appropriate and are clearly articulated. Including an additional explanation of the rational of using linear regression modeling provides more clarity in respect to the design and methodology of the study. The reporting of information is consistent with the expectations outlined in PLOS ONE.

Results:

In my opinion, the results presented by the authors were objectively presented. The authors utilized non-causal, descriptive language when discussing the results of the study. The authors acknowledged that although statistically significant differences were found to exist in between groups, the magnitude of these differences may not be significant enough to have an impact from an educational standpoint or from a clinical standpoint.

Discussion:

The Discussion section of the manuscript is very well balanced and thought out by the authors. The authors provide clear discussions on potential sources of assessment-format bias, limitations to construct validity, and the differences between confidence and competence. In addition to the previous comment, the manuscript continues to reinforce that the findings of the study should not be generalized to the real-world clinical outcomes but rather to performance on vignette-based tasks for diagnostic reasoning.

Conclusion:

The conclusion of the manuscript is concise, accurate, and presented in a moderately reserved fashion. The authors have removed any residual causal language that may still be present.

Ethics, Data Availability, and Transparency:

The authors have appropriately completed, communicated, and were compliant with all PLOS ONE guidelines regarding these sections.

Overall Assessment:

This revised manuscript has been written in accordance with the methodological, ethical, and reporting requirements of PLOS ONE, and the authors have effectively responded to the review comments and have greatly enhanced the clarity, rigor, and interpretability of their study. I would recommend that the manuscript be accepted for publication.

**********

7. PLOS authors have the option to publish the peer review history of their article (what does this mean?). If published, this will include your full peer review and any attached files.

If you choose “no”, your identity will remain anonymous but your review may still be made public.

**Do you want your identity to be public for this peer review?**  For information about this choice, including consent withdrawal, please see our Privacy Policy.

Reviewer #1: **Yes:**  Sergio Carmona

Reviewer #2: No

**********

# Editor report: Acceptance letter

**DOI:** 10.1371/journal.pone.0347129.r004

**Authors:** Renato Melo, Renato Melo

PONE-D-25-58696R1

PLOS One

Dear Dr. Maquet,

I'm pleased to inform you that your manuscript has been deemed suitable for publication in PLOS One. Congratulations! Your manuscript is now being handed over to our production team.

At this stage, our production department will prepare your paper for publication. This includes ensuring the following:

* All references, tables, and figures are properly cited

* All relevant supporting information is included in the manuscript submission,

* There are no issues that prevent the paper from being properly typeset

You will receive further instructions from the production team, including instructions on how to review your proof when it is ready. Please keep in mind that we are working through a large volume of accepted articles, so please give us a few days to review your paper and let you know the next and final steps.

Lastly, if your institution or institutions have a press office, please let them know about your upcoming paper now to help maximize its impact. If they'll be preparing press materials, please inform our press team within the next 48 hours. Your manuscript will remain under strict press embargo until 2 pm Eastern Time on the date of publication. For more information, please contact onepress@plos.org.

You will receive an invoice from PLOS for your publication fee after your manuscript has reached the completed accept phase. If you receive an email requesting payment before acceptance or for any other service, this may be a phishing scheme. Learn how to identify phishing emails and protect your accounts at https://explore.plos.org/phishing.

If we can help with anything else, please email us at customercare@plos.org.

Thank you for submitting your work to PLOS ONE and supporting open access.

Kind regards,

PLOS ONE Editorial Office Staff

on behalf of

Dr. Renato S. Melo

Academic Editor

PLOS One
